# A Simple Way to Quantify Plastic in Bats (Mammalia: Chiroptera) Using an Ultraviolet Flashlight

**DOI:** 10.3390/mps8040080

**Published:** 2025-07-17

**Authors:** Letícia Lima Correia, Ariane de Sousa Brasil, Thiago Bernardi Vieira, Magali Gonçalves Garcia, Daniela de Melo e Silva, Ana Beatriz Alencastre-Santos, Danielle Regina Gomes Ribeiro-Brasil

**Affiliations:** 1 Laboratório de Estudos de Quirópteros—LABEQ, Faculdade de Ciências Biológicas, Universidade Federal do Pará (UFPA), Campus Universitário de Altamira, Rua Coronel José Porfírio, 2515—São Sebastião, Altamira 68372-040, PA, Brazil; aryannebrasyl9733@gmail.com (A.d.S.B.); vieiratb@ufpa.br (T.B.V.); 2 Laboratório de Microbiologia, Universidade Federal do Pará (UFPA), Faculdade de Ciências Biológicas, Campus Universitário de Altamira, Rua Coronel José Porfírio, 2515—São Sebastião, Altamira 68372-040, PA, Brazil; mgarcia.bio@gmail.com; 3 Laboratory of Mutagenesis, Institute of Biological Sciences, ICB I, Federal University of Goiás, Campus Samambaia, Goiânia 74690-900, GO, Brazil; danielamelosilva@ufg.br; 4 Programa de Pós-Graduação em Zoologia (PPGZOOL), Universidade Federal Do Pará (UFPA), Av. Perimetral, 1901—Terra Firme, Belém 66077-830, PA, Brazil; ana.alencastre.aba@gmail.com; 5 Laboratório de Ecologia e Conservação de Ecossistemas Aquáticos (LECEA), Universidade Federal de Mato Grosso (UFMT), Campus Universitário do Araguaia (CUA), Av. Universitário, 3500—Universitário, Pontal do Araguaia 78698-000, MT, Brazil; danielle.brasil@ufmt.br

**Keywords:** nanoplastic, microplastics, mesoplastics, fiber, sphere, habitat monitoring, pollution, environmental contamination digestive system, respiratory system, feces

## Abstract

Bats, as key ecological players, interact with a diverse array of organisms and perform essential roles in ecosystems, including pollination, pest control, and seed dispersal. However, their populations face significant threats from habitat contamination, particularly from microplastics (MPs). This study introduces a novel, efficient, and cost-effective method for visualizing transparent microplastics using ultraviolet (UV) light. By employing handheld UV flashlights with a wavelength range of 312 to 400 nm, we enhance the detection of MPs that may otherwise go unnoticed due to color overlap with filtration membranes. All necessary precautions were taken during sampling and analysis to minimize the risk of contamination and ensure the reliability of the results. Our findings demonstrate that the application of UV light significantly improves the visualization and identification of MPs, particularly transparent fibers. This innovative approach contributes to our understanding of plastic contamination in bat habitats and underscores the importance of monitoring environmental pollutants to protect bat populations and maintain ecosystem health.

## 1. Introduction

Bats are terrestrial mammals capable of flight, feeding on nectar, fruits, seeds, insects, fish, amphibians, small mammals, and even blood [1]. Their interaction with a wide range of organisms through their diet grants them vital ecological roles. They serve as keystone species, acting as important dispersers of pioneer plant species [1,2], as both prey and predators within food webs, and in the suppression of insect pests [1]. Additionally, they play a crucial role in the pollination of plants with high economic, social, and ecological value [3], such as tequila (*Agave* sp.) [4] and pequi (*Caryocar brasiliensis*) [5]. As a result, bats are excellent bioindicators for studies aimed at assessing contamination across different habitat types.

The contamination of habitats and organisms by plastics is currently considered the primary threat to both human health and animal biodiversity [6]. Most plastics are petroleum-derived polymers, and have earned the label of “contaminants of the century” due to their global prevalence, durability, and persistence in the environment [7,8,9]. In fact, plastic contamination has already been detected in all ecosystems on the planet, causing damage in both aquatic and terrestrial environments, highlighting its significant dispersal and contamination potential [10,11]. These important flying mammals can become contaminated with plastic particles through their diet or by inhaling airborne particles [12]. Small plastic fragments, known as microplastics (MPs), which are less than 5 mm in size, can be ingested incidentally during feeding [13] or inhaled through respiration [14,15].

Ingested or inhaled MPs can accumulate in the gastrointestinal tract and respiratory pathways, potentially causing physiological effects such as altered growth, reduced survival, reproductive issues, changes in gut microbiota, and oxidative stress [16]. Plastics are classified by size into macroplastics (>25 mm), mesoplastics (<25 mm > 5 mm), microplastics (<5 mm > 1 μm), and nanoplastics (<1µm) [17]. These classifications, along with characteristics such as shape (fibers, spheres, pellets, and fragments [18]) and polymer type (e.g., PVC, PS, PP, and PET) [17], are crucial for understanding their environmental and biological impacts. Nanoplastics, in particular, are capable of crossing biological barriers and accumulating in organs such as the liver, heart, and brain [19]. Furthermore, fibers are the most commonly encountered shape in various environments [20], while PET is frequently identified as a polymer associated with adverse effects on wildlife [21]. MPs may originate primarily from being manufactured at microscopic sizes, or secondarily through the degradation of larger plastic pieces [22,23].

Bats are integral components of ecosystems, contributing to seed dispersal, pollination, and insect population control [24]. Their exposure to environmental contaminants, such as microplastics, remains poorly understood. Microplastics are pervasive pollutants found in terrestrial and aquatic systems, often entering food webs through ingestion [12]. Given the trophic diversity of bats and their reliance on different habitats, understanding the presence of microplastics in their tissues could provide critical insights into the extent and pathways of contamination in natural ecosystems.

It is important to highlight that bat conservation is directly linked to human health, and their population decline can lead to an increase in insect populations in crops, which in turn may result in the intensified use of pesticides to control pest insects [25], ultimately affecting human health [25]. Despite advances in research on microplastic contamination, there remains a significant knowledge gap in environmental monitoring studies involving mammals [12,26]. To date, only one study worldwide has provided information on the abundance, characterization, spatial distribution, and trends of microplastic contamination in flying mammals [12]. The objective of this study is to introduce a cost-effective and straightforward method for plastics in organic tissues using ultraviolet (UV) flashlights. Additionally, we present a protocol for analyzing environmental contamination by microplastics in bats. This includes outlining the capture methodology designed to minimize specimen contamination in the field, as well as a detailed approach for the quantification, characterization, and composition of microplastics in the digestive and respiratory systems, as well as in bat feces.

The work introduces a new approach for detecting microplastics in the biological tissues of bats, using a portable UV flashlight. Unlike traditional methods that require more structured laboratories with expensive equipment, this protocol is economical and easy to replicate, facilitating its application in less structured, remote laboratories with limited resources.

## 2. Experimental Design

### 2.1. Bat Sampling

We used ten mist nets (9 × 2.5 m), set up at sunset and left open for six hours, inspected every half hour to avoid bat injuries and to minimize the possibility of bats chewing and swallowing parts of the net. The captured bats were placed in 100% cotton fabric bags and transported to the laboratory—in this case, the Laboratory of Ecology of Altamira (LABECO) at the Federal University of Pará, Altamira campus. The bats were then euthanized by cervical dislocation, and morphometric data (total length, foot, ear, tragus, forearm, and weight) were recorded. Subsequently, the bats were preserved in 10% formaldehyde and stored in glass jars containing 70% alcohol. All procedures of sampling, transport, and preservation of specimens were conducted in accordance with the regulations of the Biodiversity Authorization and Information System, Instituto Chico Mendes de Conservação da Biodiversidade, Ministry of the Environment (SISBIO N° 57294-2), and the Ethics Committee on Animal Use at the Federal University of Goiás (CEUA UFG N° 004-21).

For fieldwork, researchers should preferably wear cotton clothing to avoid contaminating specimens with textile fibers from their attire. In the laboratory, the use of cotton lab coats and latex gloves is mandatory throughout the entire process.

### 2.2. Fecal Collection

After the collection process described in Section 2.1 (Bat sampling), the bottom of each bag was inspected for feces. When feces was present, they were carefully collected in a labeled paper bag corresponding to the respective bat. In the laboratory, the samples were transferred to Eppendorf tubes for further analysis.

### 2.3. Digestive Solution by Alkaline Hydrolysis

To dissolve organic matter, alkaline hydrolysis is used, employing potassium hydroxide (KOH) as the strong base to denature proteins and hydrolyze chemical bonds. Compared to sodium hydroxide (NaOH), KOH has been shown to be equally effective in dissolving organic material while causing less degradation to specific types of plastics [27,28,29]. This ensures more reliable recovery of plastic particles for subsequent analyses. The impact of KOH on polymeric materials depends on the type of plastic. Polyethylene, polypropylene, and polyamides are reported to be resistant, while polycarbonate and polyesters appear to degrade [30]. The samples of bat organs ranged from 0.0322 g for the smallest individual to 17.917 g for the largest individuals, and to dissolve the samples we used 10 mL of 10% (*v*/*v*) KOH, kept at 60 °C for 24 h. For this, we dissolved 10 g of analytical-grade KOH in 100 mL of distilled and filtered water. The procedure involved the following:

(i) Precisely weighing 10 g of KOH on an analytical balance;
(ii) Adding 100 mL of distilled and filtered water into a beaker or glass jar, and gradually incorporating the KOH.

Since preparing KOH in water is an exothermic reaction, it is recommended to mix slowly until the solution becomes completely homogeneous.

## 3. Procedure

### 3.1. Extraction of the Biological Tissues

The organs of the digestive and respiratory systems were completely excised. The digestive system was removed in its entirety, from the esophagus to the anus, while the respiratory system was extracted from the trachea to the lungs. This entire procedure was conducted within a laminar flow hood to prevent the contamination of the samples.

### 3.2. Digestion of the Tissues and Feces

The samples were placed in sanitized glass vials containing potassium hydroxide (KOH; 10%, *v*/*v*) (10 mL) to dissolve the tissues [31]. The pots with the samples were placed in an oven with a temperature of 60 °C for seven days with a protocol modified from [32] to accelerate the sample digestion process. After tissue digestion, the samples were filtered through a 0.22 µm pore size membrane of nitrocellulose (MF-millipore^TM^ and Filter type: 0.22 µm MCE Membrane 47 mm) using a vacuum pump. Although particles smaller than 50 µm were not analyzed in this study, the use of this membrane allows for future investigations of smaller particle sizes if needed. The membranes were stored in Petri dishes, protected by aluminum foil envelopes, and returned to the oven for 24 h at 60 °C for drying. Aluminum foil was used to shield the samples during drying and storage, further reducing the risk of environmental contamination.

### 3.3. Visual Analysis of Plastic Waste

The samples were analyzed using a stereo microscope with a magnification of 100 times (Digilab Trinocular Stereo Microscope DI-106T Zoom, DIGILAB acessórios para laboratórios, Piracicaba, São Paulo, Brazil). The membranes were scanned from left to right and top to bottom. Each item was photographed and identified by size, distinguishing between microplastics and mesoplastics [17]. Microplastics (MPs) are defined as having a length between 1 and 5 mm, while mesoplastics (MSPs) range in size from 5 mm to 25 cm [17].

For the identification of materials, we followed the criteria, which included the following [33]:

(a) Residues that resembled fibers with structures similar to animal joints were not considered;
(b) Only residues that maintained a consistent shape, color, and pattern along their entire structure were included;
(c) Particles smaller than 50 μm were excluded;
(d) Confirmation of plastic residues was conducted using the hot needle test [34].

### 3.4. Detection of MPs Using UV Light

**Figure 1:**
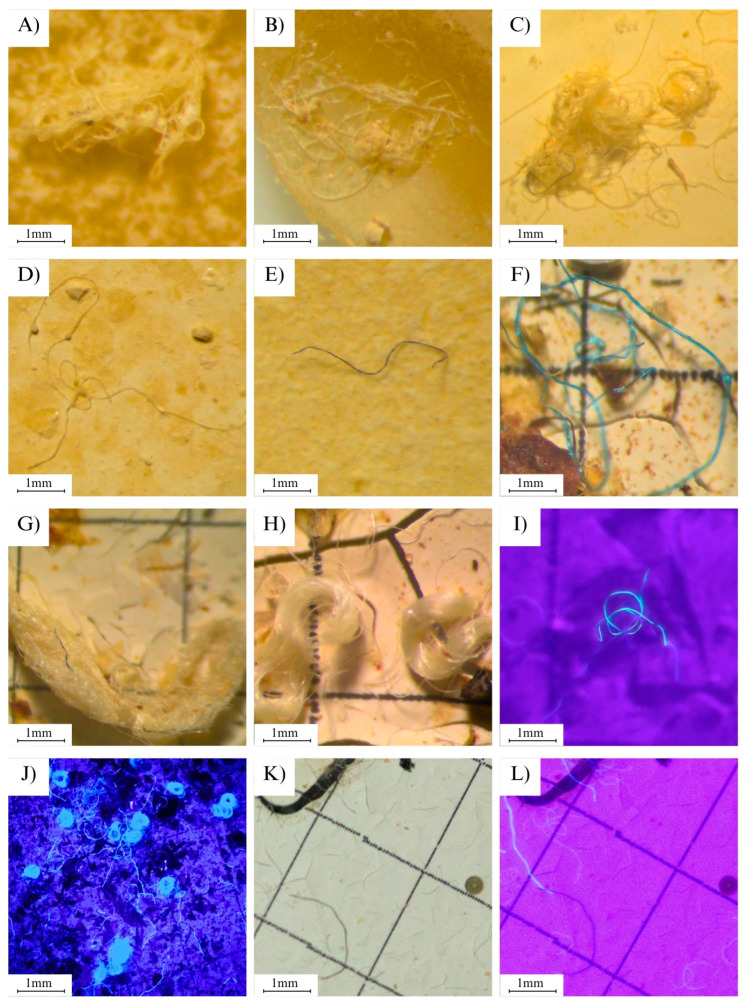
Plastics found in the digestive and respiratory systems and in the feces of bats, represented as follows: (**A**–**C**) tangles of white/transparent fibers; (**D**) white/transparent fibers; (**E**) black fibers; (**F**) blue fibers; (**G**,**H**) tangles of fibers; (**I**,**J**) fibers under UV light; (**K**) an image showing plastic fibers under magnifying light, and the same image (**L**) under UV light.

We present an efficient, innovative, and cost-effective method for visualizing transparent microplastics (MPs) using a handheld flashlight equipped with ultraviolet (UV) light in the wavelength range of 312 to 400 nm. Although this specific approach has not been previously reported in the literature, the selected wavelength range aligns with the known ability of UV light to induce fluorescence or enhance the visibility of certain polymers due to their optical and chemical properties. Transparent or white MPs can easily go unnoticed during the visualization of filtration membranes due to color overlap (i.e., a white particle on a white background). However, when illuminated with UV light, without any prior treatment, these particles become excited and subsequently emit luminescence (Figure 1J,K).

The luminescence occurs due to the fluorescence resulting from the absorption of UV light [35], leading to the excitation and emission of visible light, which often appears as a brilliant blue or pink hue. In contrast, colored MPs do not exhibit this property because the UV light is attenuated by the colored layer [36]. However, they can be more easily identified during visualization under a stereomicroscope due to color contrast.

This procedure is crucial to prevent underestimation of particle abundance. For this purpose, handheld UV LED flashlights an were used. During the process, the illumination of the stereomicroscope was turned off, and the UV flashlight was activated and directed onto the filtration membrane containing the particles. This approach enabled the identification of transparent or white particles that were otherwise invisible during standard counting under normal light. The use of handheld UV LED flashlights effectively simulates the fluorescence capabilities of specialized microscopes, providing a practical and accessible alternative for detecting microplastics.

### 3.5. Quality Assurance and Quality Control (QA/QC)

All the necessary precautions were taken for laboratory procedures, such as wearing clothes and gowns made of only 100% cotton. All analysis material was previously washed with distilled water and filtered before use. The membranes used in the filtration process were covered with aluminum foil. In addition, the amount of plastics found in the laboratory blank samples was quantified and subtracted from the results of the bat samples. This ensured that only the plastic residues originating from the bats were accounted for. For example, if the laboratory blank contained three fibers with the same characteristics as those found in the bat samples, these three fibers were subtracted from the total count of the respective bat samples [34].

## 4. Expected Results

By carefully executing all stages of the process while adhering to recommended precautions to avoid sample contamination, and completing the steps of collection, sorting, preparation, dissolution, and analysis—scanning left to right and top to bottom—it becomes possible to obtain samples similar to those shown in Figure 1, displaying various shapes and sizes. The use of UV light significantly enhances sample analysis, making it easier to visualize plastics, particularly white or transparent fibers.

Using our protocol, we successfully analyzed a total of 159 bats, focusing on both their digestive and respiratory systems. Additionally, 60 fecal samples from bats were processed, resulting in the identification of 1708 plastic particles in the digestive and respiratory systems and 12,644 particles in the fecal samples. These results highlight the importance and relevance of our protocol, demonstrating its effectiveness in advancing the study of microplastics in bats and contributing valuable insights to this emerging field. This dataset has contributed to research, including published articles, e.g., [12,37] and manuscripts currently being prepared for submission.

The methodology used in this protocol demonstrated high precision in detecting microplastics in bat tissues, as it enables the identification and quantification of their shape, type, and color. The use of UV light facilitates the visualization of white or transparent particles, which under natural light would require more time to detect. However, additional chemical analyses to identify polymer types are recommended, as they could provide complementary and detailed information on the origin and impacts of microplastics.

The protocol presented here provides an innovative and accessible method for analyzing microplastics in bats. The approach is cost-effective, user-friendly, and capable of delivering reliable results, addressing a critical gap in ecological and contamination research. Future studies should focus on refining this technique by standardizing parameters such as UV light intensity and exposure duration, as well as exploring its application to other organisms and environments.

## Data Availability

Data are available on request.
